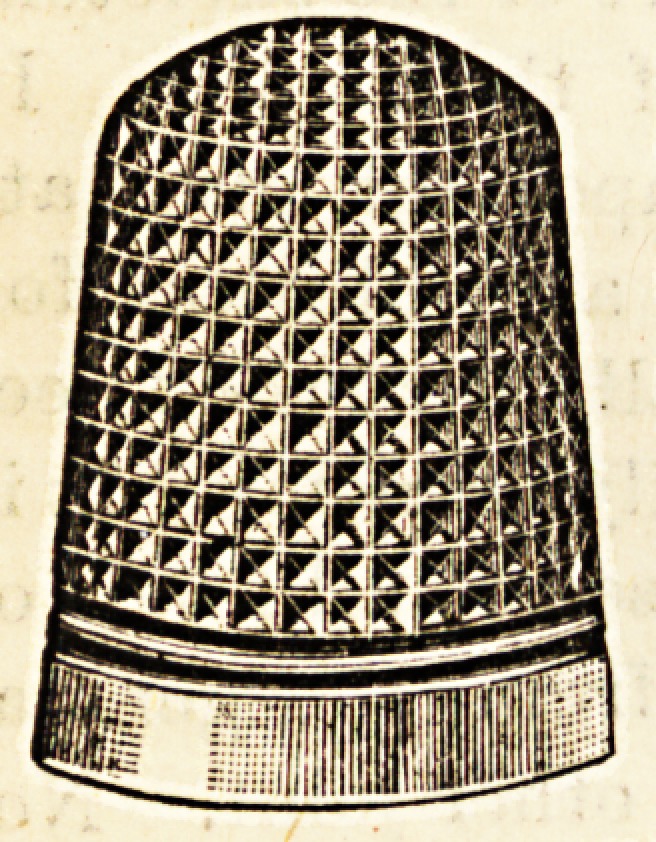# New Remedies and Appliances

**Published:** 1888-09-29

**Authors:** 


### New Remedies and Appliances.

A Boon to Ladies.—Not to all, perhaps; as we believe there are ladies who never do needlework of any kind. To such the "Dorcas" thimble will be no boon, but to the great majority it will prove itself to be just what has been wanted ever since the first needlework was done in Eden by

"The fairest of her daughters—Eve."

The "Dorcas" thimble does not wear out. The outer and inner cases are of silver and these have an inner lining of steel sandwiched between them—in fact, the thimble is armour-plated, and impenetrable and indestructible. It is, moreover, beautifully made in six sizes, and in various patterns, one of which is given in the accompanying illustration. The patentee and sole manufacturer is Mr. Charles Horner, Halifax.

A New Antiseptic.—The Patent Borax Company have put on the market a new preparation, which they call "Prepared Californian Borax." Most persons, including probably Macaulay's "Zulu," as well as his "school-boy," are acquainted with the purifying and healing properties of borax. Not all, however, are as well aware of its harmlessness as they are of its value as an antiseptic. Hitherto, it has not been easy to procure a preparation which should be at once pure, pleasant, and cheap. The Patent Borax Company guarantee the purity of their "Californian Borax," and their published statement is confirmed by Dr. Bostock Hill's report of its analysis. Of the cheapness and agreeableness of the preparation everybody can convince himself by the purchase of a penny packet. As a harmless and efficient antiseptic for the home its uses are thousandfold. It arrests decomposition, softens water, destroys bad odours, and in fact does almost everything in the way of a domestic purifier which can be required. Until something cheaper and better is discovered, it may be said with truth that every household should have it in use.

## Figures and Tables

**Figure f1:**